# Poly I: C-activated dendritic cells that were generated in CellGro for use in cancer immunotherapy trials

**DOI:** 10.1186/1479-5876-9-223

**Published:** 2011-12-30

**Authors:** Jitka Fučíková, Daniela Rožková, Hana Ulčová, Vít Budinský, Klára Sochorová, Kateřina Pokorná, Jiřina Bartůňková, Radek Špíšek

**Affiliations:** 1 Department of Immunology, Charles University, Second Faculty of Medicine and University Hospital Motol, Prague, Czech Republic; 2 Sotio, Prague, Czech Republic

**Keywords:** cancer immunotherapy, dendritic cells, Poly I:C, culture media, clinical use

## Abstract

**Background:**

For clinical applications, dendritic cells (DCs) need to be generated using GMP-approved reagents. In this study, we tested the characteristics of DCs generated in two clinical grade culture media and activated by three maturation stimuli, Poly I: C, LPS and the mixture of proinflammatory cytokines in order to identify the optimal combination of culture media and activation stimulus for the clinical use.

**Method:**

We tested DCs generation using two GMP-certified culture media, CellGro and RPMI+5% human AB serum and evaluated DCs morphology, viability and capapability to mature. We tested three maturation stimuli, PolyI:C, LPS and the mixture of proinflammatory cytokines consisting of IL-1, IL-6, TNF and prostaglandin E2. We evaluated the capacity of activated DCs to induce antigen-specific T cells and regulatory T lymphocytes.

**Results:**

Cell culture in CellGro resulted in a higher yield of immature DCs resulting from increased number of adherent monocytes. DCs that were generated in CellGro and activated using Poly I:C were the most efficient in expanding antigen-specific T cells compared to the DCs that were generated in other media and activated using LPS or the cocktail of proinflammatory cytokines. A comparison of all tested combinations revealed that DCs that were generated in CellGro and activated using Poly I:C induced low numbers of regulatory T cells.

**Conclusion:**

In this study, we identified monocyte-derived DCs that were generated in CellGro and activated using Poly I:C as the most potent clinical-grade DCs for the induction of antigen-specific T cells.

## Introduction

The aim of vaccination approaches in human cancer is to induce tumor-specific, long-lasting immune response that slows down the growth of tumor cells [1,2]. The induction of tumor immunity includes: 1) the presentation of tumor antigens by dendritic cells; 2) priming and activation of tumor antigens-specific T cells; and 3) homing of effector T cells to the tumor site and recognition of malignant cells which leads to the elimination of the tumor [3,4]. Dendritic cells are highly specialized APCs with unique capacity to establish and control primary immune responses [5]. DCs reside in peripheral tissues in an immature state where they capture and process Ag for presentation in the context of MHC molecules. After encountering appropriate stimuli, DCs differentiate into mature DCs, which are characterized by decreased endocytic activity, up-regulation of major histocompatibility complex (MHC) class I and II molecules and co-stimulatory molecules (CD86, CD80), and responsiveness to inflammatory chemokines [6]. The next generation of DC vaccines is expected to generate large numbers of high-avidity effector CD4 and CD8 T cells and to overcome regulatory T cells and the immunosuppressive environment that is established by tumors.

Circulating blood DCs are rare (they account for < 1% of human PBMC) and are difficult to maintain in culture [7]. Although in vivo expansion of blood DCs via administration of the hemopoietic growth factors Flt-3 ligand and GM-CSF can be used to increase the yield of isolated cells, most experimental and clinical studies currently rely on the in vitro development of DC-like cells from CD34+ progenitor cells or blood monocytes [8-10]. Monocytes are typically cultured for 5-7 days with GM-CSF and IL-4 to generate immature DCs that are activated for another 1-2 days with microbial, proinflammatory, or T cell-derived stimuli to obtain mature DCs with full T cell stimulatory capacity [11-14].

For clinical use, DCs must be generated using GMP-approved reagents and number of studies have addressed the need to generate large numbers of DCs for clinical trials [14-18]. In this study, we tested DC generation in two GMP-certified culture media, CellGro and RPMI+5% human AB serum and evaluated the morphology, viability and capacity to mature. DCs must be activated to efficiently activate T cells [19]. We tested three maturation signals that were available in GMP quality: PolyI:C, LPS (TLR-3 or TLR-4 ligands) and the mixture of proinflammatory cytokines (MC) consisting of IL-1, IL-6, TNF and prostaglandin E2 [20-23]. We evaluated the capacity of activated DCs to induce antigen-specific T cells and regulatory T lymphocytes.

## Materials and methods

### DC generation

Study was approved by the IRB of the Charles University, 2^nd ^Medical School. Immature monocyte-derived DCs were generated as previously described [12,24]. Briefly, peripheral blood mononuclear cells (PBMCs) were obtained from buffy coats of healthy donors after signing an informed consent and monocytes were separated by allowing 2 h of cell adhesion in 75-cm^2 ^culture flasks (Nunc). 1 × 10^7 ^adherent monocytes were cultured for 5 days in culture media in the presence of GM-CSF at the concentration of 500 IU/ml (Leukine) and 20 ng/ml of IL-4 (PeproTech)[25]. On day 5, DCs were seeded in 24-well plates (Nunc) at 5 × 10^5 ^DC/ml and activated using Poly (I:C) (Sigma-Aldrich, St. Louis, MO) at 25 μg/ml, LPS (Sigma-Aldrich) at 1 μg/ml, or a cocktail of proinflammatory cytokines (TNF, IL-1 and IL-6, PGE2). Immature and mature DCs were used for further studies.

### Flow cytometry

The following monoclonal antibodies (mAbs) against the indicated molecules were used: CD80-FITC, CD83-PE, CD86-PE-Cy5, CD14-PE-Dy590, CD8-APC (Exbio), CD11c-PE, HLA-DR-Alexa700, IFN-γ-PE, CD4-PE_Dy747 (BD Biosciences, San Jose, CA), HLA-A2 flu-MP-PE (Beckman Coulter), and FoxP3-PE (eBioscience). The cells were stained for 30 min at 4°C, washed twice in PBS+0.1% BSA and analyzed using FACSAria (BD Biosciences) with FlowJo software. DCs were gated according to their FSC and SSC properties and as CD11c-positive cells. Appropriate isotype controls and only viable DCs were included in the analysis.

### Expansion of influenza matrix peptide (MP)-specific T lymphocytes, intracellular IFNγ staining and HLA-A2-MP tetramer staining

Immature monocyte-derived DCs were activated for 4 h with the indicated stimuli: Poly (I:C) at 25 μg/ml, LPS at 1 μg/ml and a mixture of proinflammatory cytokines [IL-1β (10 ng/ml), IL-6 1000 U/ml, TNF-α (10 ng/ml and PGE2 (1 μg/ml), maturation cocktail (MC)]. Activated DCs were pulsed with HLA-A2-restricted peptide from influenza MP (GILGFVFTL) 10 μg/ml (Jpt peptide technologies, Berlin); PepMix PSA 10 μg/ml (Jpt peptide technologies, Berlin); PepMix CEF 10 μg/ml (Jpt peptide technologies, Berlin). Pulsed DCs were washed and added to 2 × 10^5 ^of autologous lymphocytes in RPMI+10% FCS in U-bottom 96 well plates at a T cell:DC ratio of 10:1 for 7 days [26]. A total of 20 U/ml of IL-2 (PeproTech EC) were added on day 3. On day 7, the lymphocytes were restimulated with fresh peptide-loaded DCs and the frequency of antigen-specific T cells was determined using intracellular staining for IFNγ. Brefeldin A (BioLegend) was added to block the extracellular release of IFNγ 1 h after restimulation. After an additional 4 h of incubation, the cells were fixed using Fixation Buffer, permeabilized with Permeabilization Buffer and stained using anti-IFNγ-PE antibody (eBioscience) and CD8-APC (Exbio). The frequency of influenza MP-specific CD8 T cells was determined by staining with HLA-A2-MP tetramers (Beckman Coulter).

### Frequency of Tregs in the peripheral blood

FoxP3+ expression in T cells was assessed using the PE-anti-human FoxP3+ Staining Kit (eBiosciences). Rat IgG2a-PE (BD Biosciences) was used as an isotype control. The samples were also simultaneously stained with CD25-Dy647 (Miltenyi Biotec), CD4-PE-Dy747, CD3-FITC. The cells were acquired using FACSAria (Becton Dickinson) and analyzed using FlowJo software (Tree Star).

### Statistical analysis

Data were analyzed by Man-Whitney non-parametric test using Statistica v.7.1 software. The results were considered statistically significant when p < 0.05.

## Results

### The yield of generated immature DCs in tested culture media

**Figure 1:**
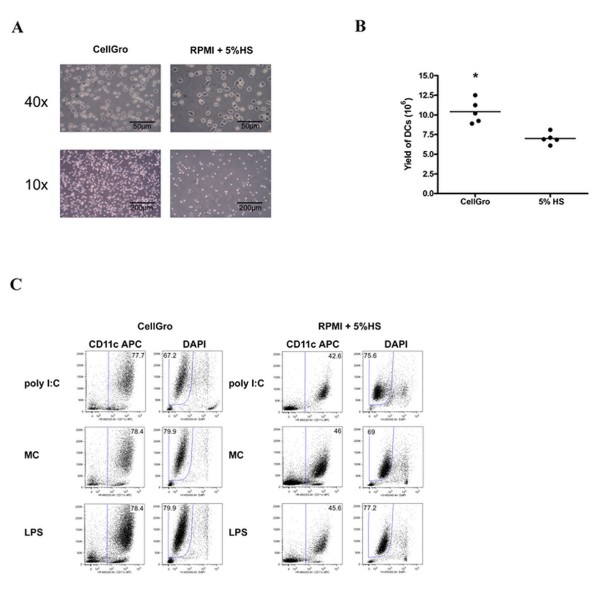
**Characteristics of immature dendritic cells that were generated in different culture media**. A. Morphology and density of adherent monocytes after 2 h of adhesion in in CellGro or RPMI+5% human AB serum. B. Total number of immature DCs that were generated from 1 × 10^7 ^of PBMCs in CellGro or RPMI+5% human AB serum. Each symbol represents an individual experiment, and the horizontal bar indicates the mean value for all experiments. * *P *value for comparison between tested conditions, *P *< 0.05. C. Purity (percentage of CD11c positive DCs) and viability (percentage of DAPI negative cells) of DCs generated in CellGro or RPMI+5% human AB serum and activated by tested maturation stimuli.

We first compared the characteristics of DCs differentiated in the tested media from 1 × 10^7 ^PBMCs. Tested media differed in the yield of monocytes after two hours of adherence. The number of adherent monocytes was significantly increased when PBMCs were cultured in CellGro medium (Figure 1A). Consequently, a significantly higher number of immature DCs was obtained after 5 days of culture in the presence of IL-4 and GM-CSF. DC generation in serum-free CellGro media yielded an average of 1,5 times more immature DCs than the culture in RPMI supplemented with 5% AB serum (Figure 1B). We did not detect any differences in cell viability and morphology between immature DCs that were generated in CellGro and RPMI+5% AB serum (Figure 1C).

### Phenotype analysis of DCs following DC maturation with different stimuli

**Figure 2:**
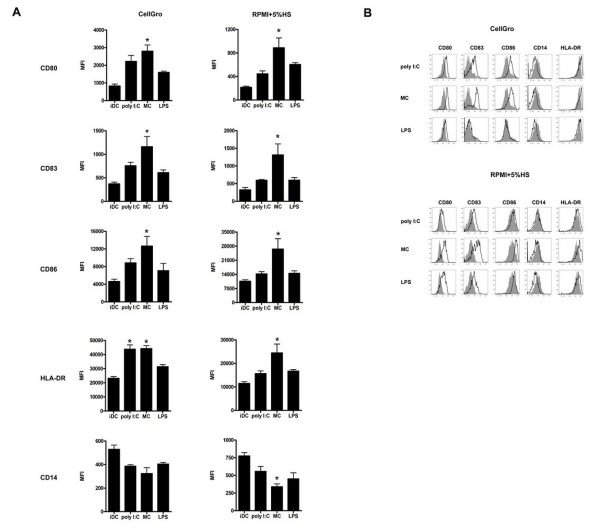
**The phenotype of dendritic cells that were generated in CellGro or RPMI+5% human AB serum and activated for 24 h using tested maturation stimuli**. The summary of six independent experiments (A) and representative histograms (B) are shown. * *P *value for comparison with immature DCs, *P *< 0.05. Grey histograms represent the expression of maturation-associated molecules on immature DCs.

To investigate the capacity of immature DCs that were generated in tested media to be activated by different maturation stimuli, we evaluated the expression of costimulatory molecules (CD80, CD83 and CD86) and antigen presentation-associated MHC class II expression (HLA-DR). As a control, immature DCs were incubated for 24 h in the presence of IL-4 and GM-CSF. Although the endocytic activity of activated DCs was not significantly different (data not shown), the phenotype analysis revealed that the tested agents induced various degrees of maturation-associated molecule expression (Figure 2). Exposure of DCs that were generated in various media to maturation signals upregulated the expression of CD80, CD83, CD86 and HLA-DR and down-regulated CD14 expression. LPS activation did not induce phenotypic maturation of immature DCs that were generated in CellGro. Treatment of immature DCs with the mixture of proinflammatory cytokines led to the most prominent changes in the expression of maturation-associated molecules.

### In vitro capacity of mature DCs to induce antigen-specific T cells

**Figure 3:**
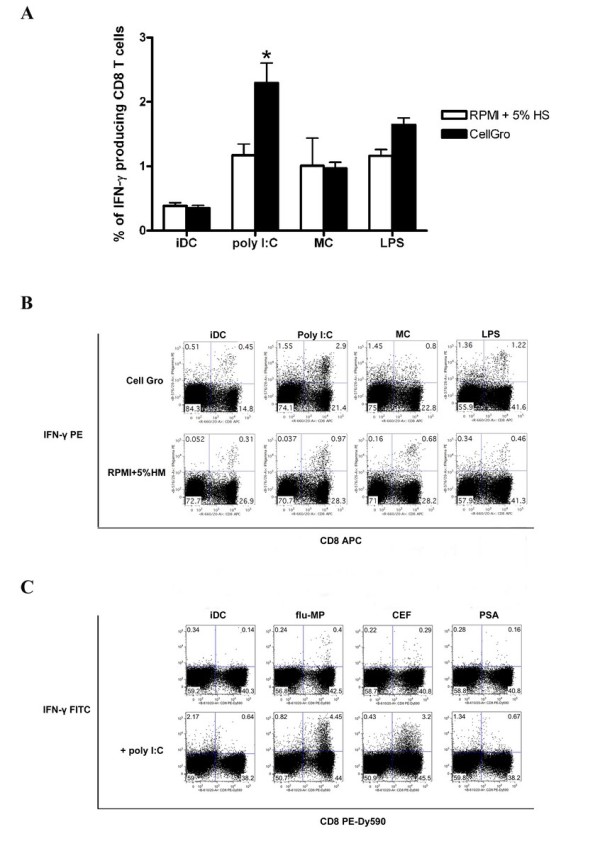
**The induction of antigen-specific T cells**. DCs that were generated in CellGro or RPMI+5% human AB serum were activated using the tested maturation stimuli and subsequently used as stimulators for the induction of antigen-specific T cells. The frequency of antigen-specific T cells was analyzed using intracellular IFNγ staining. A, B. Induction of HLA-A2 influenza MP-specific CD8 T cells. The summary (A) and representative staining (B) of five independent experiments are shown. C. Induction of HLA-A2 influenza MP-, CEF peptide mix- and PSA peptide mix-specific T cells using DCs that were generated in CellGro and activated using Poly I:C. Representative dot plots of five independent experiments are shown.

**Figure 4:**
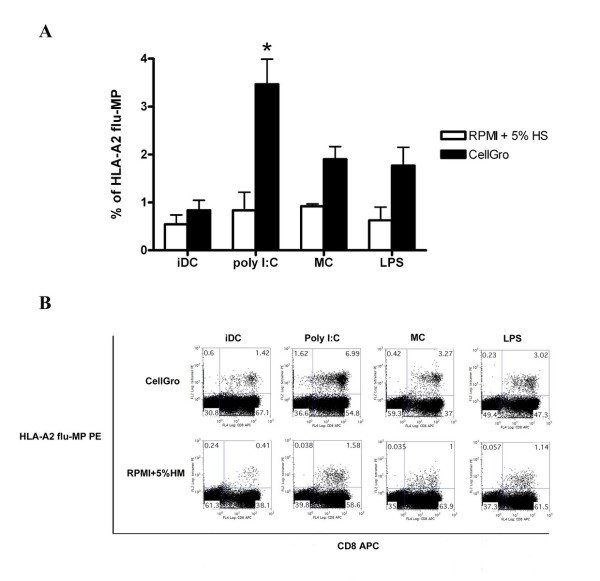
**DCs that were generated in CellGro or RPMI+5% human AB serum were activated using the tested maturation stimuli, pulsed with HLA-A2-restricted influenza MP and used as stimulators for the induction of antigen-specific T cells**. The frequency of antigen-specific T cells was determined by staining with HLA-A2 influenza MP tetramers. The summary (A) and representative staining (B) of five independent experiments are shown. The frequency of HLA-A2 influenza MP-specific T cells is shown.

Mature DCs efficiently stimulate Ag-specific T-cell responses. We evaluated the capacity of mature DCs to expand influenza matrix peptide-, CEF peptide mix- and PSA peptide mix-specific T cells. Mature DCs that were generated in various culture media were pulsed with antigens and used for the expansion of antigen-specific T cells for 7 days. The frequency of IFN-γ-producing T cells was analyzed one week later after restimulation with identical peptide-loaded DCs. DCs that were generated in CellGro and activated using Poly I:C were the most efficient in expanding antigen-specific T cells compared to DCs that were generated in other media and activated using LPS or the cocktail of proinflammatory cytokines (Figure 3). Poly I:C activated DCs generated in CellGro also efficiently expanded antigen specific T cells in other models tested in this study (influenza MP, CEF peptide mix and PSA peptide mix) (Figure 3B). Among the tested T cell populations, the highest frequency of influenza MP- specific T cells that were detected using HLA-A2 tetramer staining was seen in T cells that were stimulated using Poly (I:C)-activated DCs that were generated in CellGro (Figure 4).

### Capacity of activated DCs to induce regulatory T cells

**Figure 5:**
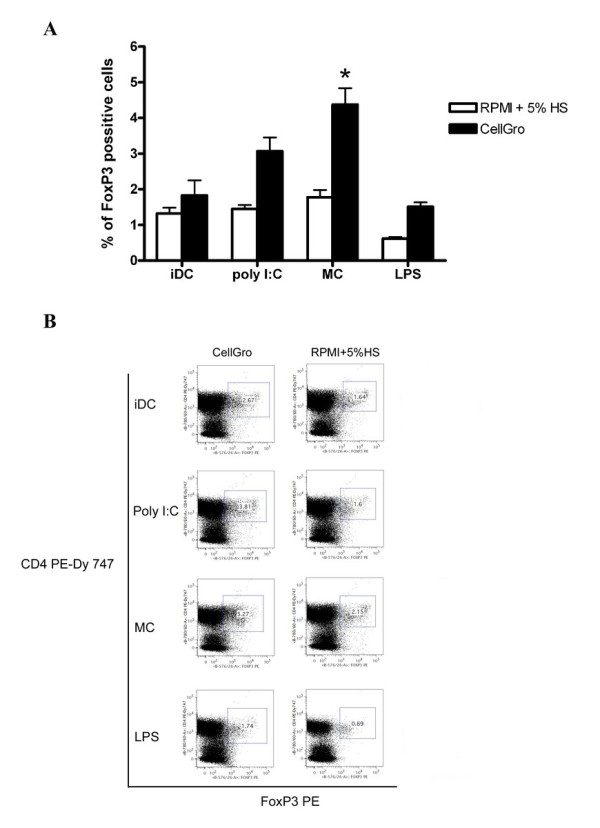
**The expansion of Tregs by DCs that were generated in CellGro or RPMI+5% human AB serum**. DCs that were generated in CellGro or RPMI+5% human AB serum were activated using the tested maturation stimuli, pulsed with HLA-A2-restricted influenza MP and used as stimulators for the induction of antigen-specific T cells. The frequency of CD4^+^CD25^+^FoxP3^+ ^cells was analyzed after 7 days of culture. The summary (A) and representative staining (B) of five independent experiments are shown. * *P *value for comparison with immature DCs, *P *< 0,05.

In the last set of experiments, we tested the capacity of DCs that were generated under various conditions to induce regulatory T cells (Tregs). Tregs expanded after 7 days of DC and T cell cocultures were determined as CD4+ CD25+ FoxP3+ T cells. DCs that were activated using a mixture of proinflammatory cytokines induced the expansion of high numbers of regulatory T cells that exceeded those induced by immature DCs. Among the different activated DC populations, Poly I:C-activated DCs that were generated in CellGro had the lowest ratio FoxP3+ Tregs/antigen specific T cells and thus appear to be the most suitable cells for use in DC cancer immunotherapy studies (Figure 5).

## Discussion

DCs have been extensively used in immunotherapy for their properties of initiators and modulators of the immune response [1]. In the immature state, DCs can be easily loaded with the desired antigens. However, immature DCs do not activate the immune response. To become efficient antigen-presenting cells, DCs must first undergo a process of maturation [5,27]. Protocols for the generation of large numbers of DCs for use in cancer immunotherapy trials need to carefully consider these characteristics of DC physiology [28-31]. Therefore, the first step of large-scale production of DCs for clinical trials is to generate large numbers of immature DCs for antigen pulsing [12,14,17,18,22,32-34]. A well-defined maturation stimulus is subsequently applied to achieve complete and reproducible maturation [12,23,29,35,36].

Current legislation for the products of advanced cellular therapies requires the use of GMP-compliant reagents and manufacturing units [2,37]. Many pioneer studies with DCs in cancer immunotherapy have been conducted with DCs that had been generated in classical complete cell culture media, i.e., RPMI that was supplemented with fetal calf or bovine sera [27,38]. The use of FBS in the current generation of DCs for cellular therapy has not been approved and therefore must be replaced by human plasma, human serum or serum-free clinical grade media. A well-defined protocol should reproducibly generate clinical-grade DCs with a stable phenotype and potent T cell stimulatory capacity. With the increasing number of clinical studies using monocytes-derived DCs for cancer immunotherapy, it is essential to provide reliable protocols to generate sufficient quantities of well-characterized, highly immunogenic DCs according to current cGMP guidelines [39,40]. In this study, we tested the characteristics of DCs that were generated using GMP-certified media (CellGro and RPMI+ 5% human AB serum), reagents and three types of maturation stimuli that were available in GMP quality. The aim was to identify the optimal combination of culture conditions that generated large numbers of clinical-grade mature monocyte-derived DCs with the capacity to induce an antigen-specific immune response. The culture of adherent monocytes in CellGro media in the presence of GM-CSF and IL-4 yielded significantly more viable immature DCs compared to that of adherent monocytes in RPMI+5% human AB serum. The difference in the final yield of immature DCs was attributed to the higher number of adherent monocytes after 2 h of adhesion in CellGro. The presence of serum slightly impeded the adherence of monocytes, which may explain the lower numbers of adherent cells in RPMI+5% human AB serum.

The maturation status of DC is crucial for adequate T cell recruitment, activation, expansion and differentiation [41,42]. Therefore, we tested the capacity of LPS, Poly I:C and a mixture of proinflammatory cytokines consisting of IL-1, IL-6, TNF and prostaglandin E2 to induce full activation of DCs that were generated in the tested clinical-grade culture media [2,21,22,43]. Among the tested conditions, the maturation of DCs with the mixture of proinflammatory cytokines resulted in the highest expression of maturation-associated molecules in CellGro and in RPMI+5% human AB serum. LPS and Poly I:C induced comparable maturation-associated changes in RPMI+5% human AB serum. However, LPS was a weak inducer of DC maturation in serum-free CellGro media most likely due to the absence of LPS-binding protein. The ability to activate antigen-specific T cells is critical for the clinical effect of DC-based vaccines [44-46]. Therefore, we used HLA-A2-restricted influenza MP as a model antigen to test the activating potential of generated DCs. Among all of the tested conditions, DCs that were generated in CellGro and activated using Poly I:C were the most efficient in inducing influenza MP-specific T cells based on frequency and function, which were determined by assessing influenza MP tetramer staining and IFNγ production, respectively [47-49]. Poly I:C-activated DCs that were generated in CellGro efficiently expanded T cells that were specific for the peptide mix consisting of influenza, CMV- and EBV-derived peptides and the PSA peptide mix [50,51]. Furthermore, Poly I:C-activated DCs that were produced in CellGro induced significantly lower numbers of regulatory T cells compared to DCs that were activated using the cytokine cocktail. This result is in accordance with recently published data showing that cytokine-matured DCs expand Foxp3-positive inhibitory Tregs in vitro and in vivo [52,53].

## Conclusions

In this study, we demonstrated that monocyte-derived DCs that were generated in CellGro and activated using Poly I:C potently induced antigen-specific T cells. This protocol has been approved for clinical use by regulatory authorities and represents a platform for the manufacturing of DC-based active cancer immunotherapy in the settings of prostate and ovarian cancer.

## Abbreviations

DC: dendritic cells; APC: antigen-presenting cell; Poly (I, C): Polyriboinosinic polyribocytidylic acid; PAMPs: pathogen-associated molecular patterns.

## Competing interests

The authors declare that they have no competing interests.

## Authors' contributions

JF carried out experiments and helped to draft the manuscript. DR carried out experiments. HU carried out experiments, organized donor database. VB carried out flow cytometry experiments and analysis. KS KP carried out the stimulations of antigen specific T cells. JB helped to design the study. RS conceived and designed the study and drafted the manuscript. All authors read and approved the manuscript.
